# Consensus on the application of negative pressure wound therapy of diabetic foot wounds

**DOI:** 10.1093/burnst/tkab018

**Published:** 2021-06-21

**Authors:** Shizhao Ji, Xiaobin Liu, Jie Huang, Junmin Bao, Zhaohong Chen, Chunmao Han, Daifeng Hao, Jingsong Hong, Dahai Hu, Yufeng Jiang, Shang Ju, Hongye Li, Zongyu Li, Guangping Liang, Yan Liu, Gaoxing Luo, Guozhong Lv, Xingwu Ran, Zhongmin Shi, Juyu Tang, Aiping Wang, Guangyi Wang, Jiangning Wang, Xin Wang, Bing Wen, Jun Wu, Hailin Xu, Maojin Xu, Xiaofei Ye, Liangxi Yuan, Yi Zhang, Shichu Xiao, Zhaofan Xia

**Affiliations:** 1 Burn Institute of PLA, Department of Burns, The First Affiliated Hospital of Naval Medical University, No. 168 Changhai Road, Yangpu District, Shanghai, 200433, China; 2 Fujian Burn Institute, Fujian Medical University Union Hospital, No. 29 Xinquan Road, Gulou District, Fuzhou, 350001, China; 3 Department of Burns & Wound Care Center, Second Affiliated Hospital of Zhejiang University, College of Medicine, No. 88 Jiefang Road, Shangcheng District, Hangzhou, 310009, China; 4 No. 3 Department of Burns and Plastic Surgery and Wound Healing Center, The Fourth Medical Center of Chinese PLA General Hospital, No 51 Fucheng Road, Haidian District, Beijing, 100048, China; 5 Foot and Ankle Surgery Department, Guangzhou Zhenggu Orthopedic Hospital, No. 449 Dongfeng Middle Road, Yuexiu District, Guangzhou, 510031, China; 6 Department of Burns and Cutaneous Surgery, The First Affiliated Hospital of Air Force Medical University, No. 127 West Changle Road, Xincheng District, Xi’an, 710032, China; 7 Wound Healing Department, PLA Strategic Support Force Characteristic Medical Center, No. 9 Anxiang North Lane, Chaoyang District, Beijing, 100101, China; 8 Department of Peripheral Vascular, Beijing University of Chinese Medicine, Dongzhimen Hospital, Hai Yun Cang on the 5th, Dongcheng District, Beijing, 100700, China; 9 Department of Orthopedics, Zhejiang University School of Medicine, Sir Run Run Shaw Hospital, No. 3 East Qinchun Road, Shangcheng District, Hangzhou, 310016, China; 10 Department of Burns, The Fifth Hospital of Harbin, No. 27 Jiankang Road, Xiangfang District, 150030, Harbin, China; 11 Institute of Burn Research, State Key Laboratory of Trauma, Burn and Combined Injury, Southwest Hospital, Third Military Medical University, Gaotanyan Street no. 29, Shapingba District, Chongqing, 400038, China; 12 Department of Burn, Shanghai Jiaotong University, School of Medicine Affiliated Ruijin Hospital, No. 197 Ruijin Road (No.2), Huangpu District, Shanghai, 200025, China; 13 Department of Burn Surgery, the Third People’s Hospital of Wuxi, No. 585 North Xingyuan Road, Wuxi, 214043, China; 14 Innovation Center for Wound Rpair, Diabetic Foot Care Center, Department of Endocrinology and Metabolism, West China Hospital, Sichuan University, No. 37 Guoxue Lane, Chengdu, China; 15 Department of Orthopedics, Shanghai Jiaotong University Affiliated Sixth People’s Hospital, No. 600 Yishan Road, Xuhui District, Shanghai, 200233, China; 16 Department of Hand and Microsurgery, Xiangya Hospital of Central South University, No. 87 Xiangya Road, Kaifu District, Changsha, 410008, China; 17 Diabetic Foot Centre, The Air Force Hospital From Eastern Theater of PLA, Nanjing, No.1 Malu Road, Qinhuai District, 210002, China; 18 Department of Orthopedic Surgery, Beijing Shijitan Hospital, Capital Medical University, No. 10 Tieyi Road, Haidian District, Beijing, 100038, China; 19 Department of Plastic and Hand Surgery, Ningbo No. 6 Hospital, No. 1059 East Zhongshan Road, YinZhou District, Ningbo, 315040, China; 20 Plastic and Burn Surgery Department, Diabetic Foot Prevention and Treatment Center, Peking University First Hospital, No.8, Xishiku Street, Xicheng District, Beijing, 100034, China; 21 Department of Burn and Plastic Surgery, Second People’s Hospital of Shenzhen, Shenzhen University, No. 3002 West Sungang Road, Futian District, Shenzhen, 518037, China; 22 Department of Orthopedics and Trauma, Peking University People’s Hospital, Peking University, No.11 Xizhimen South Street, Beijing, 100044, China; 23 Diabetic Foot Treatment Center, Peking University People’s hospital, Peking University, No.11 Xizhimen South Street, Beijing, 100044, China; 24 Department of Burn and Plastic Surgery, Affiliated Hospital of Nantong University, No. 20 Xisi Road, Nantong, 226001, China

**Keywords:** Vacuum sealing drainage, Vacuum-assisted closure, Vacuum-assisted therapy, Negative pressure wound therapy, Topical negative pressure therapy, Suction wound closure therapy, Diabetic foot, Diabetic ulcer, Diabetic wound

## Abstract

Because China is becoming an aging society, the incidence of diabetes and diabetic foot have been increasing. Diabetic foot has become one of the main health-related killers due to its high disability and mortality rates. Negative pressure wound therapy (NPWT) is one of the most effective techniques for the treatment of diabetic foot wounds and great progress, both in terms of research and its clinical application, has been made in the last 20 years of its development. However, due to the complex pathogenesis and management of diabetic foot, irregular application of NPWT often leads to complications, such as infection, bleeding and necrosis, that seriously affect its treatment outcomes. In 2020, under the leadership of Burns, Trauma and Tissue Repair Committee of the Cross-Straits Medicine Exchange Association, the writing group for ‘Consensus on the application of negative pressure wound therapy of diabetic foot wounds’ was established with the participation of scholars from the specialized areas of burns, endocrinology, vascular surgery, orthopedics and wound repair. Drawing on evidence-based practice suggested by the latest clinical research, this consensus proposes the best clinical practice guidelines for the application and prognostic evaluation of NPWT for diabetic foot. The consensus aims to support the formation of standardized treatment schemes that clinicians can refer to when treating cases of diabetic foot.

Highlights

- Irregular application of NPWT often leads to complications, and seriously affect its treatment outcomes.
- The writing group for the present article was established with the participation of scholars from the specialized areas of burns, endocrinology, vascular surgery, orthopedics and wound repair.
- The consensus aims to support the formation of standardized treatment schemes that clinicians can refer to when treating cases of diabetic foot.

## Background

Diabetic foot is a serious complication in patients who have advanced diabetes and refers to foot infections, ulcers and/or deep tissue destruction caused by nerve abnormalities and vascular lesions in the distal lower limb(s) of these patients. According to the International Working Group on the Diabetic Foot [1], an amputation for diabetic foot is performed every 20 seconds and for more than 1 million people every year. In 2017, there were 425 million diabetic patients globally and this is expected to increase to 629 million by 2045. According to an expert opinion published in the New England Journal of Medicine, 19–34% of diabetic patients develop diabetic foot ulcers, the mortality rate 5 years post amputation is greater than 70% and the mortality rate 2 years post amputation in those on kidney dialysis is as high as 74% [2]. The mortality risk in patients with diabetic foot is much higher than in those with some malignant tumors. Diabetic foot has become one of the main health-related killers due to its high disability and mortality rates; therefore, its prevention and treatment has become an urgent clinical issue.

The treatment of diabetic foot requires a cross-disciplinary and systematic approach that comprises blood glucose control, surgical debridement, vascular recanalization, decompression treatment and supportive treatment. Controlling wound infection and promoting tissue repair are vital for preventing amputation or reducing the level of amputation [3, 4]. The concept of negative pressure wound therapy (NPWT) was first established and applied in clinical practice by a German physician, Fleischmann, in 1993 and has, ever since, been recognized for its remarkable effect in improving wound drainage, enhancing perfusion and promoting the growth of granulation tissue. Today, NPWT is widely used for various acute and chronic wounds, such as diabetic foot ulcers. In 2016, NPWT had been recommended with class I evidence by the Wound Healing Society of the United States in its diabetic foot ulcer treatment guidelines. NPWT improves wound healing by reducing edema, removing bacterial products and approximating wound edges and should be considered as a treatment strategy when others fail [5]. In 2017, the European Wound Management Association had reported that NPWT-assisted treatment for diabetic foot ulcers promotes granulation tissue proliferation and accelerates wound healing [6]. The International Working Group on the Diabetic Foot recommended using NPWT to promote ulcer healing in its 2019 international guidelines on the prevention and management of diabetic foot [1].

As NPWT is an important adjunctive treatment for diabetic foot wounds, it should be standardized in terms of its application conditions, parameter adjustments, evaluations and other important aspects. The consensus committee of Tucson Expert Consensus Conference on V.A.C. Therapy first published ‘Guidelines regarding negative wound therapy (NPWT) in the diabetic foot’ in 2004 in the United States [7]; this document, which was later updated and revised in 2006, provides suggestions on the clinical application of NPWT for diabetic foot wounds [8]. Although NPWT was developed within 30 years, rapid progress in research and its clinical applications has been made, especially regarding its use in the management of diabetic foot wounds.

For these reasons, and for the purpose of establishing evidence-based guidelines, the Tissue Repair of Burns and Trauma Committee and the Cross-Straits Medicine Exchange Association discussed the latest clinical research and drafted this ‘Consensus on the application of negative pressure wound therapy for diabetic foot wounds’. By providing guidance on the best clinical practice guidelines for the application and prognostic evaluation of NPWT for diabetic foot, this consensus aims to support the formation of standardized treatment schemes that clinicians can refer to when treating cases of diabetic foot.

## Methods

### Data retrieval

This consensus was compiled by professional scholars from the specialized areas of burns, endocrinology, vascular surgery, orthopedics and wound repair and based on high-quality literature on the application of NPWT for diabetic foot. This article is based on domestic and foreign guidelines, combined with the clinical experience and research results and is written with an emphasis on practicability and feasibility. Each recommendation in this article represents a consensus from the specialists committee. However, due to the lack of sufficient evidence from large-scale randomized controlled trials (RCTs) and a comprehensive understanding from the specialists committee, many of the recommendations in this paper are preliminary and require further evidence before they can be fully recommended.

The key words used in the search for relevant literature were: vacuum sealing drainage, vacuum-assisted closure, vacuum assisted therapy, NPWT, topical negative pressure therapy, suction wound closure therapy, diabetic foot, diabetic ulcer and diabetic wound. The search was conducted on the PubMed, Embase and Cochrane Library databases. The retrieval time ranged from the time the databases were established to 1 July 2020. References to support manual retrieval were also used for topics limited to human diseases. The types of articles that were reviewed included meta-analyses, systematic reviews, randomized controlled trials, retrospective series reviews, clinical case series and expert panel recommendations.

### Levels of evidence and grades of recommendation

The levels of evidence and grades of recommendation of each article were classified according to a modified version of the evaluation system of Oxford Centre for Evidence-based Medicine levels of evidence (May 2001) (Table 1) [9]. According to the Delphi technique, each evidence guideline underwent independent and repeated evaluations by every member of our panel of experts before a unanimous opinion regarding recommendations was reached.

**Table 1:** Level of evidence and grade of recommendation for the application of NPWT in DFU

**Level of evidence**	
Strong	Based on well-designed randomized controlled trials, meta-analyses or systematic evaluations
Moderate	Based on well-designed cohort or case–control studies
Weak	Based on well-designed case series and expert advice
**Grade of recommendation**	
Strong	Indicating a clear treatment effect or highly unanimous recognition by experts
Moderate	Indicating unclear possible risks and effect after treatment
Weak	Indicating expert opinions about a likely treatment choice with low level of evidence at the present stage

This table is modified based on 2001 Oxford Centre for Evidence-Based Medicine ‘Levels of Evidence’ and ‘Grades of Recommendation’ [9]*NPWT* Negative Pressure Wound Therapy, *DFU* Diabetic Foot Ulcer

## Recommendations

### Application of NPWT for diabetic foot wounds

**Overview** The treatment of diabetic foot wounds requires a multi-disciplinary and systematic approach [10, 11]. The therapeutic goal should be to not only prevent the spread of infection and reduce the level of amputation but also prevent the progression of systemic atherosclerotic diseases, delay the occurrence of diabetes-related complications, prevent the occurrence of cardiovascular and cerebrovascular events and reduce mortality [12]. For diabetic foot wounds, the fundamental therapeutic principles are the control of wound infection, improvement of local tissue perfusion and promotion of tissue repair. Due to its excellent effects of enhancing local perfusion, promoting granulation tissue growth and improving wound healing, NPWT has become an important adjuvant treatment in the management of diabetic foot wounds [13–25]. According to many clinical randomized controlled studies, the application of NPWT for diabetic foot wounds may significantly increase the rate of wound healing, shorten healing time and reduce the rate of amputation [26–38]; it is currently recommended by the Wound Healing Society and the European Wound Management Association [39–41].

(*Grade of recommendation: strong; level of evidence: strong.*)

**Prerequisite conditions** NPWT for diabetic foot wounds is recommended under the following conditions:

(1) Wound infection is well-controlled: after debridement, the necrotic tissue has cleared and infection (especially if hidden in the fascia or interstitial space) has been controlled [42–49].
(2) The risk of bleeding is well-controlled: bleeding has completely stopped after debridement, there is no active bleeding or exposed vascular damage on the wound, there is no serious risk of coagulation dysfunction or potential bleeding and INR range from 1.0 to 2.0 [7, 50, 51].
(3) The risk of ischemia is well-controlled: there is adequate wound and distal limb perfusion and TcpO2 > 40 mmHg, ABI range from 0.9 to 1.3 or TBI ≥ 0.6 [52–58].

(*Grade of recommendation: moderate; level of evidence: moderate.*)

**Parameter settings**  *Pressure* For diabetic foot ulcers without vascular lesions, the recommended pressure range is between −125 and −80 mmHg [59, 60]. For vascular stenotic or occlusive lesions, the recommended pressure range is between −80 mmHg and −60 mmHg [61].

*Mode* NPWT may be applied in a continuous, intermittent or variable mode. In clinical practice, the most commonly used mode is continuous, in which a stable level of negative pressure is maintained. Intermittent pressure therapy (IPT) is a relatively newer mode in which the negative pressure device switches on and off at preset time intervals (e.g. 5 minutes on, 2 minutes off). Many studies have shown that the blood flow and granulation tissue growth achieved by the IPT mode are much better than those seen when using the continuous mode. However, due to the tissue deformation that occurs with every cycle of IPT, patients may experience significant pain. Therefore, the variable pressure therapy (VPT) mode was created. The biggest difference between the VPT and IPT modes is that the minimum negative pressure value of the VPT mode is a certain negative pressure value (e.g. −10 mmHg), rather than 0 mmHg. The VPT mode has the same advantages as the IPT mode but causes less pain and is easily accepted by patients. Both preclinical and clinical studies have shown that IPT and VPT modes lead to better granulation tissue growth, wound contraction and wound epithelialization. It is therefore recommended that the continuous mode is applied for the first 48 hours, followed by the IPT (5 minutes on, 2 minutes off) or VPT mode (high: −80 mmHg, low: −10 mmHg).

*Note* The settings should be adjusted according to the patient’s condition and the size of the wound [62–65].

(1) For larger and more complex wounds that are difficult to seal, the negative pressure value may be increased accordingly.
(2) For patients who have undergone skin grafting or dermal stent grafting, the continuous mode should be applied for 5–7 days.
(3) For patients who are at risk of bleeding (e.g. coagulation dysfunction or long-term anticoagulant use), the negative pressure value should be reduced accordingly.
(4) Some patients cannot tolerate even slight amounts of pain; therefore, the NPWT mode should be selected according to the patient’s pain tolerance.

(*Grade of recommendation: moderate; level of evidence: moderate.*)

**Evaluation and management of the effect of NPWT in treating diabetic foot wounds** For surgical debridement of diabetic foot wounds, the ‘nibbling’ principle, with limited batched debridement, is usually adopted. There are an abundance of soft tissue and fascial spaces in the feet. In clinical practice, a small amount of necrotic tissue may remain within the wound and, even after several rounds of debridement, it is unlikely that hidden foci of infection would have been removed. Therefore, there is a risk that infection may spread after NPWT [66]. In addition, even after revascularization of the ischemic wound, there is a short-term risk of reclosure of blood vessels and wound ischemia. At the same time, for patients who need to take anticoagulants for a long duration, there may be a risk of wound bleeding after NPWT [67]. In order to detect potential risks like infection, bleeding, ischemia, it is recommended that daily evaluation is done to carefully inspect for wound pain, redness and swelling; changes in skin color and temperature around the wound; and color, odor and volume of wound drainage fluid; along with blood tests and imaging to comprehensively evaluate for wound infection, ischemia, bleeding, and the overall condition of the patient [68–71]. If wound infection is not under control, avascular necrosis is aggravated or the wound continues to bleed, the negative pressure dressings should be removed and the wound should be reevaluated. NPWT may be applied again only after infection is controlled, tissue ischemia has improved and the risk of bleeding has reduced [72]. If pain and swelling are aggravated, but without wound infection, tissue ischemia, or other systemic conditions, it is recommended to reduce or suspend the negative pressure, change the mode of negative pressure treatment for observation and remove the negative pressure if necessary [73].

After 1–2 rounds of NPWT application, a comprehensive evaluation of its effects should be conducted. The effectiveness evaluation and recommended treatment measures are as follows [74–77].

(1) Significantly effective: there is growth of new granulation tissue on the wound surface or a reduction of the wound surface with surrounding epithelialization; it is recommended to continue NPWT.
(2) Effective: wound infection or tissue ischemia improves, the wound is ruddy and blood perfusion is good; it is recommended to apply NPWT 1–2 times and re-evaluate.
(3) Ineffective: wound infection or tissue ischemia does not improve, the infection is aggravated or the tissue is more necrotic; it is recommended to stop the NPWT, recanalize the blood vessels and re-evaluate after infection is controlled.

(*Grade of recommendation: moderate; level of evidence: moderate.*)

**Frequency of replacement** The frequency of NPWT replacement after debridement should be determined based on the condition of the wound [78–81]. In the absence of infection, active bleeding or tissue ischemia, it is recommended that the dressing is replaced after 3–5 days and within 7 days. The frequency of dressing replacement after skin grafting may be extended to 5–7 days.

*Note* Polyvinyl alcohol foam is hydrophilic, so it tends to harden and block the vessels when there is less wound exudation and may even compress the wound site and cause tissue ischemia; thus, close observation and timely NPWT dressing replacement is required. The pore diameters of polyurethane foam are relatively large, providing room for granulation tissue to grow into. Therefore, NPWT should not be applied for a long time to avoid the risk of granulation tissue growing into the porous structure of the foam and causing damage and unnecessary blood loss during foam removal [82].

(*Grade of recommendation: weak; level of evidence: weak.*)

**Common complications and their management** Before applying NPWT for diabetic foot wounds, the application conditions should be fully understood. Continuous assessment should occur throughout the process and the application should be stopped or the vacuum dressings replaced according to the situation of the wounds [83–85]. Compared with conventional diabetic foot wound treatment methods, NPWT does not significantly increase complications. The possible complications and recommended measures are as follows.

(1) In cases of wound bleeding or aggravation of wound infection, treatment with NPWT should be stopped immediately, the NPWT device should be removed and the conditions should be reassessed after the infection is controlled by hemostasis or debridement and a dressing change [86].
(2) In cases of aggravation of ischemia or necrosis, treatment with NPWT should be stopped immediately, the NPWT device should be removed and the conditions should be reassessed after the ischemia improves by increased perfusion [87].
(3) In cases of eczema around the wound or tension vesicles on the region of normal skin under the applied film (the most common complications), a skin film should be applied to protect the surrounding skin, the vacuum pressure should be reduced and skin stretch when applying the film should be minimized [88].
(4) In cases of granulation tissue growing into the foam material, NPWT should not kept for too long and be replaced at intervals of 3–5 days. Foam material should be removed as thoroughly as possible to prevent a secondary infection [89].
(5) If the wound pain or edema worsens, but wound infection, tissue ischemia and systemic conditions can be excluded, negative pressure might be reduced or paused, the negative mode can be changed and closely observed, or remove it if necessary [90].

(*Grade of recommendation: moderate; level of evidence: weak.*)

**Economic evaluation** Compared to conventional treatment, NPWT reduces the number of dressing changes, the consumption of medical supplies and the requirement of human resources; this results in lower treatment expenses and medical costs and a higher overall potency ratio. Therefore, NPWT is recommended for the treatment of diabetic foot wounds [91–105].

(*Grade of recommendation: moderate; level of evidence: moderate.*)

**Application of improved NPWT** In recent years, many improved NPWTs have been used for diabetic foot wounds, including NPWT with instillation [64, 106–114] and local oxygen negative pressure [115]. Preliminary studies have shown that these have certain advantages in preventing and controlling wound infections and promoting wound debridement [107, 116–118]; however, there is limited high-quality research evidence at present and large-scale multicenter clinical verification is still needed.

(*Grade of recommendation: weak; level of evidence: weak.*)

### Specific applications of NPWT for complicated diabetic foot wounds

**Infected wounds** Diabetic foot wounds are highly susceptible to infection and, if severe, may develop into extensive cellulitis, osteomyelitis or even necrotizing fasciitis, which are life-threatening. For severely infected patients, a comprehensive assessment of the patient’s systemic condition is required and amputation should be performed if necessary [119–121]. For limb-salvage patients, multiple debridement operations should be performed. Vascular recanalization and other treatments may be used to improve distal tissue ischemia and ensure sufficient blood perfusion after necrotic tissue removal and infection control [122–125]. NPWT may only be applied on this basis. At the same time, continuous assessment is required during the application and NPWT should be stopped or replaced according to the clinical situation [126–128].

(*Grade of recommendation: moderate; level of evidence: weak.*)

**Wound exposing bone and/or tendon** For wounds exposing bone and/or tendon, it is recommended that a skin flap coverage be used. NPWT may be used to improve the basic condition or cultivate granulation tissue to create a suitable condition for the transfer of a skin flap or skin graft in the following cases.

(1) The basic conditions of the wound are currently unsuitable for skin flap repair [129–131].
(2) Skin flap repair cannot be performed due to vascular stenosis or poor blood supply. [132].
(3) Small wounds exposing bone and/or tendon [96, 133].

(*Grade of recommendation: weak; level of evidence: weak.*)

**Wounds with osteomyelitis** For diabetic foot wounds with osteomyelitis, thorough debridement, removal of sequestrums and systemic antibiotic therapy for 2–4 weeks are required. NPWT should be used until the infection is effectively controlled [134–136]. Continuous assessment and close observation for local infection of the wound are required during the application process and NPWT should be stopped or replaced accordingly [137–139]. For osteomyelitis patients with poorly controlled infection, or in cases for whom the control of infection is yet to be determined, NPWT should be used with caution [140, 141].

(*Grade of recommendation: moderate; level of evidence: weak.*)

**After skin graft or flap transfer** Studies have shown that for both reticular skin grafts and stamp skin grafts, continuous vacuum suction effectively increases the survival rate of the skin and shortens the time required for wound healing [142–145]. It is recommended that NPWT is applied in continuous vacuum suction mode with a pressure between −100 and −80 mmHg for 5–7 days according to wound exudation [146–148].

The conventional use of NPWT devices is not recommended after flap transfer. If NPWT is used, it is necessary to avoid pressing the pedicle of the flap. Continuous vacuum suction should be performed for 48 hours, followed by IPT (suction for 5 minutes, pause for 2 minutes) with a pressure between −80 and −60 mmHg for 3–5 days according to wound exudation [149, 150].

(*Grade of recommendation: moderate; level of evidence: weak.*)

**Wounds grafted with dermal equivalents** Studies have confirmed that the NPWT device effectively promotes vascularization of the dermal equivalent. At the same time, its good drainage effect may prevent fluid accumulation under the dermal equivalent and its decreased need for frequent dressing changes may reduce possible wound exposure and infection, improve the success rate of dermal equivalent grafting and provide good conditions for subsequent skin grafting [151–154]. After dermal equivalent grafting, continuous vacuum suction mode should be applied with a pressure between −100 and −80 mmHg [155, 156] for 5–7 days according to wound exudation.

(*Grade of recommendation: moderate; level of evidence: weak.*)

**Wounds after extremity or toe amputations (with condition of primary suture)** NPWT is recommended due to its good drainage effect, which reduces the accumulation of exudate in the stump, and for its fixation effect (due to vacuum sealing for stabilizing the stump tissue), which promotes tissue remodeling and wound closure [157–159]. Continuous vacuum suction mode should be applied with a pressure between −80 and −60 mmHg for 5–7 days according to wound exudation.

(*Grade of recommendation: moderate; level of evidence: weak.*)

**Wounds after extremity or toe amputations (without condition of primary suture)** NPWT is recommended for the treatment of stump wounds to promote granulation tissue proliferation and tissue reconstruction after the risks of ischemia and bleeding are reduced, necrotic tissue is cleared and infection is controlled [160, 161]. The IPT (suction for 5 minutes, pause for 2 minutes) or VPT mode should be applied, with pressures between −80 mmHg and −60 mmHg or −80 mmHg and −10 mmHg, respectively, for 3–5 days according to wound exudation.

(*Grade of recommendation: strong; level of evidence: strong.*)

## Conclusion

The treatment of diabetic foot requires a cross-disciplinary and systematic approach, within which NPWT is an important adjunct treatment for diabetic foot wounds. The standardized management and application of NPWT may improve wound exudate drainage, enhance blood perfusion and promote wound healing. In view of the potential risks after its application, we compiled this consensus through a systematic review of the literature, aiming to form standardized treatment schemes for diabetic foot through the use of NPWT. However, it is necessary to point out that the levels of evidence in this consensus are not very high; therefore, more high-quality randomized controlled trials are needed to determine the most appropriate application methods and potential effects of NPWT for diabetic foot wounds.

## Abbreviations

IPT: intermittent pressure therapy; NPWT: negative pressure wound therapy; VPT: variable pressure therapy.

## Funding

Research on in situ skin repair and regeneration based on micro-tissue engineering technology and 3D printing. (The National Key R&D Program of China, Grant Number 2019YFA0110503). The study on natural living micro-amniotic scaffolds to dynamic regulate immune inflammation and reconstruct wound repairing. (National Natural Science Foundation of China, Grant Number 81971836). The systemic study of miR-23b_24–1 cluster in the prevention and treatment of MODS caused by sepsis after burns. (National Natural Science Foundation of China, Grant Number 81930057). The experimental study on regulating the immune inflammatory microenvironment of burn wounds and promoting repair and regeneration based on micro-tissue engineering technology. (National Natural Science Foundation of China, Grant Number 81871559).

## Conflicts of interest

There were no commercial interests between any of the members of the working group during the compilation process. Each member of the working group made a statement regarding his/her conflict of interest.
